# 3D Printing in Biocatalysis and Biosensing: From General Concepts to Practical Applications

**DOI:** 10.1002/asia.202400717

**Published:** 2024-11-07

**Authors:** Jonathan Nyenhuis, Christopher Heuer, Janina Bahnemann

**Affiliations:** ^1^ Institute of Physics Chair of Technical Biology University of Augsburg Universitätsstr. 1 Augsburg 86159 Germany; ^2^ Institute of Physics Centre for Advanced Analytics and Predictive Sciences University of Augsburg Universitätsstr. 1 Augsburg 86159 Germany

**Keywords:** 3D Printing, Biocatalysis, Biosensors, Rapid Prototyping, Surface Functionalization

## Abstract

3D printing has matured into a versatile technique that offers researchers many different printing methods and materials with varying properties. Nowadays, 3D printing is deployed within a myriad of different applications, ranging from chemistry to biotechnology –including bioanalytics, biocatalysis or biosensing. Due to its inherent design flexibility (which enables rapid prototyping) and ease of use, 3D printing facilitates the relatively quick and easy creation of new devices with unprecedented functions.. This review article describes how 3D printing can be employed for research in the fields of biochemistry and biotechnology, and specifically for biocatalysis and biosensor applications. We survey different relevant 3D printing techniques, as well as the surface activation and functionalization of 3D‐printed materials. Finally, we show how 3D printing is used for the fabrication of reaction ware and enzymatic assays in biocatalysis research, as well as for the generation of biosensors using aptamers, antibodies, and enzymes as recognition elements.

## Graphical abstract

This article gives an overview about 3D printing for the fabrication of biosensing and biocatalytic devices. We discuss advantages and challenges of the most used 3D printing techniques and methods for surface treatment of 3D‐printed polymers as well as specific applications in respective fields to provide an insight about important steps to be considered for device fabrication.

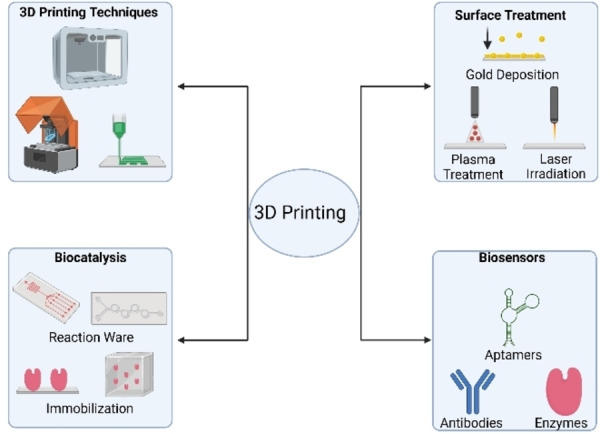

## 1 Introduction

In recent years, additive manufacturing techniques – collectively known as 3D printing – have gained widespread recognition as a highly promising technology with many applications in biotechnology ranging from cell culture assays[^1^, ^2^] and bioprocess technology^[3]^ to bioanalytics,[^4^, ^5^] biosensing[^6^, ^7^, ^8^] and biocatalysis.[^9^, ^10^] The fundamental concept underlying 3D printing is to envision all three‐dimensional objects as the culmination of their individual 2D elements, which can then be progressively stacked upon each other. A commonly used design file format for this purpose is the Standard Tessellation Language (.STL), which represents an object‘s surface geometry through a series of triangles.^[11]^ These design files can easily be created using computer‐aided design (CAD) software and directly fabricated by 3D printers that operate with minimal need for expertise.

In combination with recent improvements in resolution and speed, these technological advancements now allow state‐of‐the‐art 3D printer systems to serve as rapid prototyping platforms, where many advantageous material properties are available (translucence, heat resistance, electrical conductivity, and biocompatibility).[^12^, ^13^, ^14^, ^15^, ^16^] This straightforward and versatile process contrasts with traditional methods like PDMS‐based soft lithography, which require unique master molds for each design.^[17]^ Therefore, 3D printing offers a far more efficient and versatile approach for the fabrication of devices suitable for different applications in biocatalysis, biosensors and bioanalytics.

The term “3D printing” is really an umbrella term, however, and it now encompasses a wide range of different manufacturing methods – including stereolithography (SLA), dynamic light processing (DLP),^[18]^ extrusion‐based (EP) and fused deposition modelling (FDM),^[19]^ various inkjet‐based techniques (IJP) such as MultiJet printing (MJP),^[20]^ and even emerging techniques like Binder Jetting (BJ)^[21]^ or Aerosol Jet Printing (AJP).[^22^, ^23^]

Each method has its distinct advantages and disadvantages, leading to areas of preferred use in the research fields of biocatalysis and biosensors. As such, microfluidic flow cells and reactors for biocatalytic[^24^, ^25^] and bioanalytic applications,[^26^, ^27^] where precise control of small fluid volumes or fluid mixing is required are created by SLA and DLP (creation of exceptionally small channels[^28^, ^29^]) or inkjet‐based techniques (creation of complex internal 3D structures).[^30^, ^31^]

On the other hand, FDM printers have a lower resolution but offer low‐cost production^[32]^ with a wide range of biocompatible thermoplastic polymers.[^19^, ^33^, ^34^] One of its application fields lies in the generation of electrochemical biosensors,[^35^, ^36^] as well‐established thermoplastics can be printed together with conductive 3D printing materials such as carbon black. Emerging 3D printing techniques allow the fabrication of porous microfluidic channels for immunoassays, where liquid samples are driven through the system by capillary force (BJ)^[37]^ or offer the printing of conductive patterns and functional components on various substrates for bioelectrode fabrication (AJP).[^38^, ^39^]

**Figure 1:**
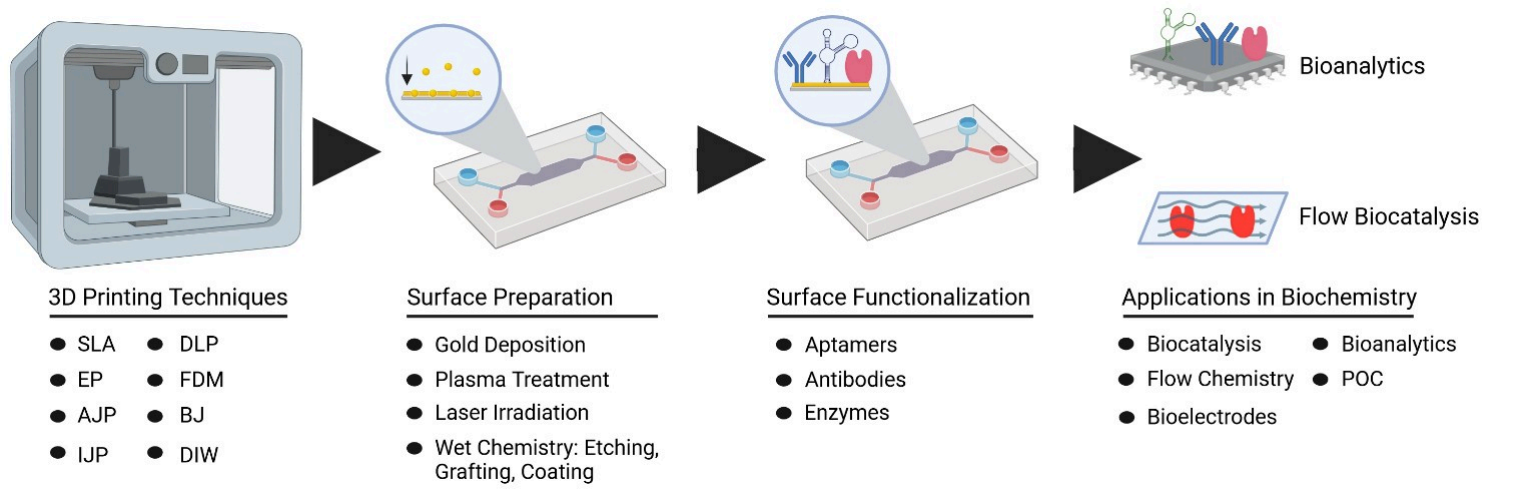
Workflow for the development of a 3D‐printed microfluidic device with surface functionalization. After choosing the respective 3D printing technique, the surface of the obtained device can be prepared by various surface activation techniques, followed by functionalization with different biomolecules for applications in bioanalytics or biocatalysis.

In this review, we will provide an overview of biocatalytic and biosensing systems generated with the help of (or in combination with) 3D printing, focusing primarily on work that has been published since 2020. We will also discuss potential options and process steps for the development of functionalized microfluidic devices (see Figure 1), including various 3D printing techniques, cutting‐edge methods for surface preparation, functionalization of 3D‐printed materials, and applications in biocatalysis and biosensor design.

## 2 3D Printing Techniques

A variety of different 3D printing techniques have been deployed for biochemistry research (*e. g*., biocatalysis and bioanalytics) in recent years. An overview is presented in Table 1. For instance, 3D printing was used to produce reaction ware,^[55]^ as a platform for enzyme immobilization^[56]^ or the generation of 3D‐printed bioelectrodes.^[57]^ Special materials like conductive carbon,^[15]^ polymer composites,[^16^, ^58^] or graphene^[59]^ – which are often used within the context of multi‐material 3D printing^[60]^ – have been well received by researchers in recent years. Additionally, various 3D printing techniques such as photopolymerization[^42^, ^61^] or FDM[^19^, ^33^] have been extensively covered in the recent literature, where detailed information about these respective processes can be found. Therefore, for the ease of the interested reader, the following chapter aims to provide merely a brief overview of the primarily 3D printing techniques that are relevant to modern biocatalysis and biosensor research. As many other reviews have already been published that seek to detail the characteristics, advantages, and potential disadvantages associated with various materials and different 3D printing technologies, however, we would also refer the reader to those sources for additional reading.[^30^, ^31^, ^61^]

**Table 1:** Overview of 3D printing techniques used for applications in biocatalysis and biosensing.

3D Printing Technology	Materials	Resolution	Advantages	Disadvantages
Stereolithography (SLA)	Proprietary UV‐curable photopolymers^[31]^	~10 μm[^40^, ^41^]	Simple and scalable, smooth surfaces, high resolution.^[42]^	Low mechanical strength, removing uncured resins is difficult, only printing of straight layers possible, slower compared to FDM.^[42]^
Dynamic‐Light‐Processing (DLP)	Photopolymers, ceramics^[43]^	<10 μm^[43]^	Higher speed compared to SLA, reuse of uncured photopolymers possible, low‐cost printers.^[44]^	Not ideal to print large structures, difficult to control precise structural shape, high cost of materials.^[43]^
Extrusion‐based Printing (EP)/Fused‐Deposition‐ Modelling (FDM)	ABS, PLA, PC, PS, graphene/conductive‐carbon[^35^, ^36^, ^45^]	>50 μm^[46]^	Wide range of materials, faster than SLA, combination of polymers with conductive materials, useful for the generation of bioelectrodes.[^35^, ^36^, ^45^]	Lower resolution compared to SLA, self‐prepared materials can have low strength^[47]^
Inkjet‐based Printing	Proprietary UV‐curable polymers[^48^, ^49^] carbon‐nanotubes^[50]^	~50 μm^[51]^	High resolution, high speed, easy setup, generation of complex structures due to removable supporting materials,^[52]^ only proprietary materials available^[53]^	Limitation in mechanical and functional properties,^[53]^ limited layer thickness^[52]^
Binder Jetting (BJ)	Metal powders, ceramics, polymer powders^[54]^	~50 μm^[54]^	Compatibility with many powdered materials, operates at r. t. under atmospheric pressure, no need for supporting structures, capable of producing complex geometries.^[54]^	multi‐step process (post‐processing steps), higher surface roughness and lower resolution compared to SLA or DLP.^[54]^
Aerosol Jet Printing (AJP)	Metal, polyaniline, PDMS, carbon‐nanotubes, graphene.^[23]^	~10 μm^[23]^	No support materials needed, wide range of materials, high resolution, able to produce multilayer patterning of conductive, dielectric/semiconducting materials, printing on nonplanar surfaces.^[23]^	High cost for special inks and printing equipment, limited to low‐viscosity inks.^[23]^

Stereolithography (SLA) represents the very first 3D printing technique ever developed (in the 1980s). In this technique, a photopolymer is selectively cured or solidified utilizing a UV laser source (photopolymerization).^[62]^ A stage or carrier plate is immersed in a bath containing this photopolymer alongside a photoinitiator, and its Z position can be gradually adjusted to define the printing height of each layer. Given that the laser must cure every spot, the printer‘s resolution is bound by the minimum pixel size of the laser beam.^[62]^ Channel dimensions below 30 μm have been achieved using this method, as documented in the literature.^[63]^ SLA is renowned for its capacity to generate highly detailed and precise models with smooth surfaces, but it is often comparatively slower than other modern 3D printing techniques. An additional enhancement aimed at addressing the low printing throughput and speed of SLA is the introduction of digital light processing (DLP), which enables the simultaneous curing of all relevant spots within a layer.^[18]^ It is important to note that since DLP technology relies on a digital light projector, the resolution of each layer is fundamentally dependent upon and limited by the projector‘s pixel density. Apart from commercialized materials, a wide array of self‐defined formulations of biocompatible and transparent materials can also be used as printing materials with SLA and DLP.[^42^, ^61^] Applications in biochemistry research include (for instance) the production of reaction ware such as microfluidic flow cells^[64]^ or micromixers.^[49]^

Inkjet‐based 3D printing is another popular technology in many application fields.[^52^, ^65^] For example, this technology is frequently employed to generate microfluidic flow cells for biosensors.^[48]^ Both the primary and support materials are dispensed drop by drop through printheads containing an array of nozzles. The overall resolution depends on the size of the droplets.^[42]^ Nevertheless, this method enables the creation of structures in the range of hundreds of micrometers and smaller.[^65^, ^66^, ^67^] The main material is usually comprised of a proprietary acrylate, which is subsequently cured using UV light.[^68^, ^69^] The support material serves the purpose of enabling the fabrication of overhanging and intricate 3D structures by filling voids and cavities, such as microfluidic channels – but it is important to note that this support material must be removed once the printing process is finished.[^48^, ^49^] Several commercial suppliers provide a diverse selection of materials with varying characteristics, such as rigidity, flexibility, transparency, biocompatibility, and high‐temperature resistance.[^12^, ^70^, ^71^]

Powder‐based 3D printing techniques like binder jetting (BJ), which has its origins in metal powder technology,^[21]^ have also been employed to develop microfluidic immunoassays.^[72]^ In BJ, a powdered material is evenly spread layer by layer onto a surface and then selectively fused together by using a binder to form a solid 3D structure.^[54]^ BJ offers several advantages, including compatibility with many powdered materials, operability at room temperature under atmospheric pressure, and no need for supporting structures even though it is capable of producing complex geometries.^[54]^ However, BJ is a multi‐step process (post‐processing steps) which results in printed parts with lower relative density. Additionally, higher surface roughness and lower resolution are attained compared to other 3D printing techniques, such as SLA or DLP.^[54]^

Aerosol Jet Printing (AJP) is an advanced additive manufacturing technology that was initially pioneered in the field of microelectronics.^[11]^ Recently, it has begun to attract significant interest in the fields of biotechnology and biochemistry research, especially for its usefulness in creating biosensing systems.^[39]^ AJP utilizes atomization and a stream of inert gas to deposit inks as focused aerosol sprays which are then directed towards the printer plate. This emergent technology enables the non‐contact deposition of functional liquid inks with a resolution close to 10 microns. Its ability to facilitate multilayer patterning of conductive, dielectric, and semiconducting materials, along with its ability to print on nonplanar surfaces, highlights the versatility that makes it a compelling option. Additionally, the advantages of digital patterning for rapid prototyping and small‐volume production render it suitable for a wide range of applications, such as sensor generation.^[38]^

Fused Deposition Modeling (FDM) is a straightforward extrusion‐based method which relies on a heated printhead in combination with thermoplastic polymers. The polymers are melted and extruded onto a surface, then cooled and solidified.^[19]^ Unlike other methods that require external support materials to fill voids, FDM uses delicate support structures which are printed simultaneously with the main structure, thus enabling the creation of overhanging elements.^[30]^ FDM also provides researches with the freedom to choose from a variety and combination of low‐cost and easily accessible materials, such as acrylonitrile butadiene styrene (ABS),^[73]^ polyethylene terephthalate glycol (PETG),^[74]^ polylactic acid (PLA),[^74^, ^75^, ^76^] and polyurethane (PU),^[17]^ among many others. In the context of biochemistry research, FDM has been used to produce reaction ware^[77]^ and biosensing electrodes.^[35]^ Limitations include the use of heat‐sensitive materials, the potential risk of fluid leakage, and challenges in printing integrated channels, however.^[30]^ Additionally, FDM typically results in higher surface roughness and lower printing resolution when compared to other surveyed techniques.[^68^, ^78^]

Other extrusion‐based methods do not require the heating of thermoplastic polymers. Instead, they rely on the extrusion of polymers through a nozzle under mechanical pressure to form a continuous filament.^[79]^ This filament is then deposited layer by layer, followed by solidification to fabricate a construct with the desired features and properties. The extruded filament is positioned at the designated location by the movements of the XYZ‐axis to create the desired patterns. Once a single layer is completed, either the extrusion head ascends or the platform descends to facilitate the deposition of the subsequent layer. These sequential steps are then repeated until the intended object is fully fabricated.^[79]^

Direct ink writing (DIW) is an extrusion‐based printing technique designed for crafting intricate 3D structures at the meso‐ and microscale. It utilizes driven deposition of a viscoelastic ink through a fine nozzle onto a computer‐controlled translational stage, enabling the construction of various scaffolds and 3D constructs. After extrusion, the 3D construct solidifies, generating a structure of desired features and properties. DIW offers remarkable versatility, facilitating the creation of multi‐material structures by separate and simultaneous extrusion of different ink materials. As a result, the use of DIW can potentially save on manufacturing time, energy, cost, and waste, while simultaneously preserving crucial material properties, when compared to inkjet writing (which is limited to the use of UV‐curable inks).^[80]^

## 3 Surface Preparation and Functionalization in 3D Printing

**Figure 2:**
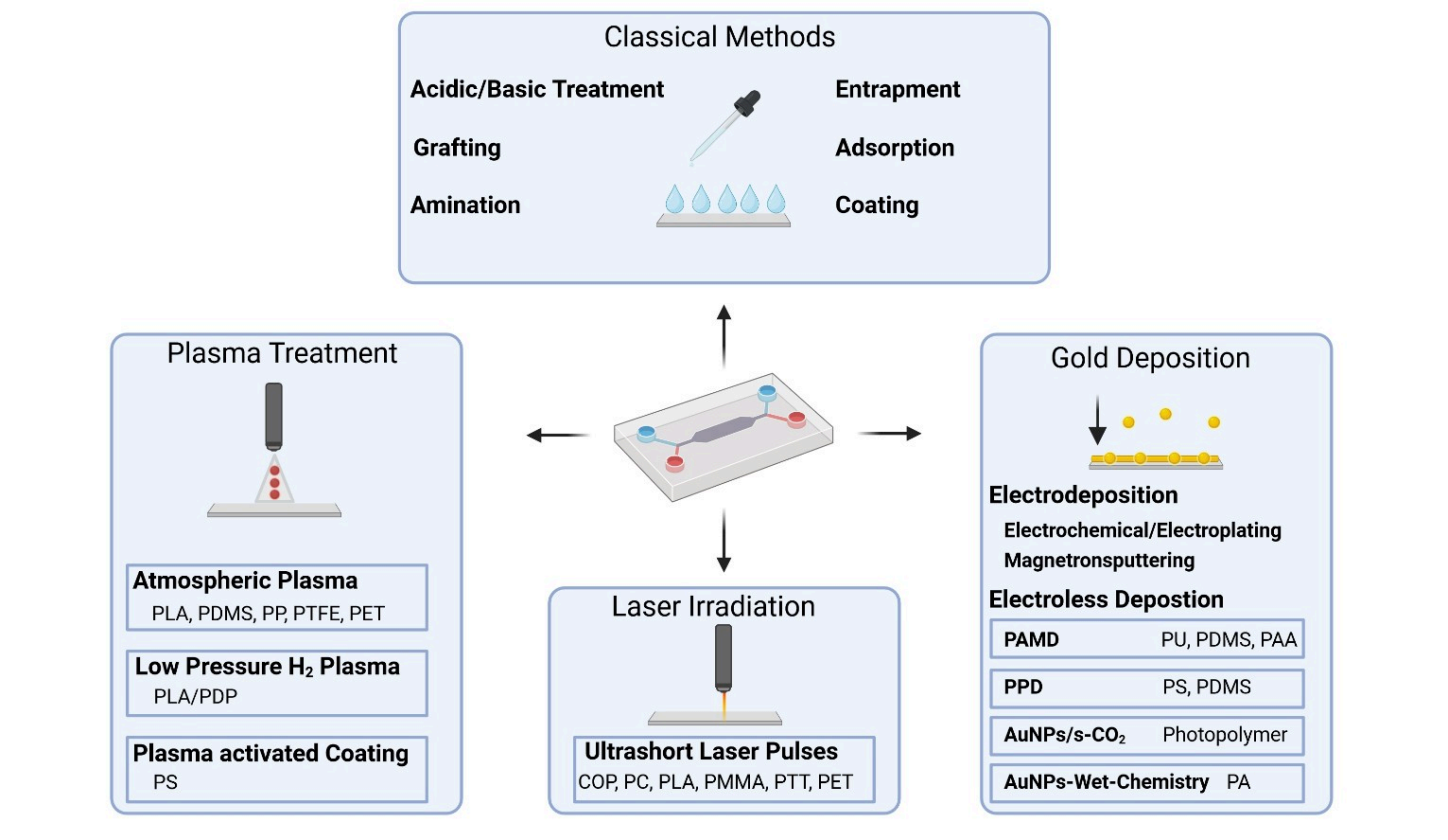
Overview about classic and newly emerged surface preparation techniques. The respective techniques and subcategories (highlighted) are shown together with the respective polymers to which they were applied. Also, classical methods are summarized, without the respective polymers, since these methods have been used for a wide range of materials.

Surface functionalization of 3D‐printed materials plays a vital role in various microfluidic applications such as flow biocatalysis (e. g., for enzyme immobilization), biosensor development, and point‐of‐care (POC) diagnostics (e. g., for the immobilization of capture elements like antibodies and aptamers). The surface properties of commonly used polymers like PLA or polycarbonate (PC) can easily be adapted to fit specific requirements through the use of different approaches (see summary in Figure 2). Traditional techniques for surface functionalization include unspecific immobilization of biomolecules such as proteins by physical adsorption^[81]^ or entrapment. More specific functionalization can be achieved by chemical treatment with acids or bases to generate hydroxyl^[82]^ or carboxyl groups.^[83]^ These functional groups can then be exploited for amination (introduction of amine groups) via EDC/NHS chemistry^[83]^ and subsequent functionalization with biorecognition elements like antibodies, enzymes, or aptamers. Moreover, various coating,^[84]^ etching,^[85]^ and grafting techniques^[86]^ are available to prepare the 3D‐printed surface for further immobilization of biomolecules. These traditional techniques have already been reviewed in detail in recent years.[^87^, ^88^] Therefore, in this chapter, we will instead describe in more detail emerging technologies for surface preparation and functionalization – like gold deposition, plasma treatment, and laser irradiation (depicted in Figure 2). These techniques originated outside of the fields of life sciences/biochemistry, but they are expected to greatly impact the use of 3D‐printed materials in all research fields where surface functionalization is required.

Gold deposition is a surface activation technique that is often used for biosensors because of its biocompatibility, conductivity, and straightforward functionalization with biomolecules such as aptamers,^[89]^ antibodies[^90^, ^91^] or enzymes.^[92]^ Gold can be either deposited via electrodeposition (e. g., electroplating,^[93]^ magnetronsputtering^[94]^) or by electroless approaches. Electrodeposition involves the creation of metallic clusters on solid surfaces. These clusters are generated by the reduction of gold cations in an electrolyte‐containing metal salt solution (typically HAuCl_4_) which is achieved by applying a potential.^[95]^ The respective substrate serves as the cathode (negative electrode) within an electrolytic cell, while the anode (positive electrode) is represented by the metal which is plated or an inert conductive material. The specific morphologies obtained depend on the electrochemical parameters employed during the process.^[95]^ In general, though, applying traditional gold deposition techniques from the semiconductor industry on 3D‐printed polymers is challenging because these polymers often exhibit dimensional instability towards vacuum, heat, or organic solvents.^[96]^ For this reason, various multi‐step methods relying on different principles for electroless gold deposition on polymer substrates have been developed in recent years.

For instance, Yu et al. developed a technique called polymer‐assisted metal deposition (PAMD), which uses a thin functional polymer interface layer to facilitate the electroless deposition (ELD) of thin metal films on various organic polymers, including PU, PDMS and poly acrylic acid (PAA).^[96]^ First, the surface is modified with anchoring polymers. Subsequently, catalytic moieties (metal nanoparticles) are immobilized onto the polymer layers to create areas that support the electroless deposition of the respective metal. Advantages of PAMD include strong adhesion between the metal and the substrate at their interface because of their interpenetration. The functional interface layer also ensures that metal deposition can occur on a wide array of soft polymer substrates. The disadvantages of PAMD are the limited choice of metals due to the respective ELD chemistry, and the limited resolution for nano‐electric applications caused by the lateral diffusion of the catalyst during ELD.^[96]^

Kim et al. developed a highly efficient three‐step route for electroless gold plating on 3D‐printed polyacrylate (PA) plastics.^[97]^ Their process relies on the electrostatic interaction between the modified negatively charged surface and the positively charged gold nanoparticles (AuNPs). The first step includes a wet‐chemistry‐based (acid‐catalyzed) hydrolysis of the polymer to introduce negatively charged −COOH groups on the surface. Then, positively charged AuNPs are seeded, followed by electroless plating of the substrate using an aqueous mixture of HAuCl_4_ and NH_2_OH*HCl.^[97]^

Other researchers have also employed nanoparticle/polymer‐assisted photochemical (PPD)^[98]^ or nanowire‐based^[99]^ approaches for electroless gold deposition on various polymers. For example, Cheng et al. applied supercritical CO_2_ (s‐CO_2_) for the electroless plating of gold on 3D‐printed polymers.^[100]^

Plasma treatment is yet another surface activation technique that has recently found its way into biochemical/biotechnological applications to improve the wettability of 3D‐printed polymers and prepare these surfaces for functionalization with biomolecules. When using plasma as a surface activation technique, however, several different factors must be considered. For one thing, the use of low‐temperature plasma (low usually referring to the temperature of the electrons^[101]^) is preferred when the thermal degradation of polymers or preexisting material components should be avoided.^[102]^ For example, low‐temperature hydrogen plasma was used to enhance the oxidative self‐polymerization of polydopamine (PDP) soaked PLA surfaces.^[103]^ PDP functioned as the anchor substance for key surface functionalization with catechins, amines, and imines, allowing further immobilization of biomolecules.^[103]^

Atmospheric pressure plasma (AP) offers the advantage that no special reaction vessel is needed, since the required pressure corresponds to the atmospheric pressure.^[104]^ AP has been used with different compositions for the activation of various polymers such as PDMS,^[105]^ PLA,^[106]^ polypropylene (PP),^[107]^ or polytetrafluoroethylene (PTFE)^[108]^ for subsequent biofunctionalization. For instance, Bilek et al. employed AP for the covalent immobilization of bovine serum albumin (BSA) on PTFE surfaces.^[108]^ Their process relies on surface‐embedded radicals, generated by plasma immersion ion implantation. Subsequently, these radicals were utilized to covalently immobilize biomolecules without the need for additional reagents.^[108]^

To introduce functional groups that can be further employed for the immobilization of biomolecules such as enzymes or antibodies, the plasma composition is important. In general, exposure to oxygen‐containing plasmas (e. g., CO_2_, O_2_, air) leads to the formation of hydroxy, peroxide, and carboxy groups,[^105^, ^109^] while nitrogen‐containing plasmas (e. g., N_2_, NH_3_, N_2_/H_2_) are used to form amino and amide groups on the polymer surface.^[110]^ For example, Duran et al. used plasma‐generated carboxy groups on PLA to covalently bind NH_2_‐containing biomolecules such as peptides and proteins.^[106]^ The plasma‐based generation of hydroxyl, carboxyl, and amino groups was also employed to immobilize the enzyme glucose oxidase (GOx) onto polyester^[111]^ and bioluminescent enzymes on microfibrous polyethylenterephthalat (PET) non‐wovens without any substantial loss of enzymatic activity.^[112]^

In another study, Gleize et al. developed a reagent‐free plasma method – namely, plasma‐activated coating (PAC) – to prepare polystyrene (PS) microplates for one‐step immobilization of DNA and streptavidin.^[113]^ In PAC, a mixture of gases (including a carbon‐containing gas) was used to generate a plasma‐activated coating on the substrate surface. PAC shows great potential for the immobilization of antibody‐binding molecules (streptavidin) without the need for additional linkers, pointing towards an easily accessible way for the development of colorimetric or fluorescence immunoassays.

In recent years, ultrashort impulse laser irradiation has also emerged as a non‐contact and highly selective process for the modification of various 3D‐printed polymers. In general, electrons of the polymer are excited by laser photons, resulting in the generation of heat which then alters the properties of the respective material.^[114]^

One parameter which is often impacted by laser irradiation is the surface wettability of the respective polymer. Wettability changes can be useful for the generation of microfluidic devices, e. g., in enhancing fluid transport through microfluidic channels due to increased hydrophilicity of the material. Surface wettability and, accordingly, the water contact angle (WCA) can both be adjusted through the introduction of hierarchical and periodic structures on the polymer surface.[^115^, ^116^, ^117^] This technique has been successfully deployed using various 3D‐printed polymers in recent years, including cyclic olefin polymers (COP),^[118]^ PC,[^115^, ^116^] PLA,^[119]^ Poly (methylmethacrylate) PMMA,^[120]^ poly (trimethylene terephthalate) PTT,^[121]^ and PET.^[117]^

The energy of laser irradiation can also be used to activate surfaces. Park et al., used a CO_2_‐laser for the chemically grafting of polyethylenimine (PEI) on PMMA.^[120]^ Successful production of amide compounds (e. g., the reaction of the amine groups of PEI with the ester groups of PMMA) was observed, and they resulted in increased hydrophilicity (reduced WCA) of the hydrophobic PMMA surface. Such surface modifications can facilitate the non‐specific adsorption of biomolecules including enzymes, peptides, etc. on 3D‐printed polymers.^[122]^ Laser ablation has also been used in combination with SLA/PLA to fabricate reaction ware like passive micromixers.^[123]^ In the field of biosensing, laser‐induced graphene (LIG) has attracted more and more interest in recent years as a platform for the generation of sensor platforms. By subjecting flexible carbon‐rich polymers – such as polyimide (PI) – to pulsed laser irradiation, the sp3‐carbon atoms can be induced to undergo photothermal conversion to sp2‐carbon fractions, resulting in the formation of graphene sheets.^[124]^

LIG‐based electrochemical biosensors, in combination with enzymes (GOx), have been developed for the detection of glucose[^125^, ^126^, ^127^, ^128^] as well as aptamer/oligonucleotide‐based systems for thrombin^[129]^ and miRNA^[130]^ detection. Additionally, LIG‐based systems have been utilized for the simultaneous detection of metabolites (ascorbic acid (AA), dopamine (DA) and uric acid (UA).^[131]^

## 4 3D Printing for Biocatalysis

Biocatalysis is considered to be a key tool for transforming chemical reactions in our collective push towards pursuing a more sustainable future, often referred to as “green chemistry”. Enzymes can catalyze a broad range of chemical transformations under mild conditions, often with high selectivity. Not surprisingly, their use in various industrial, pharmaceutical, and environmental processes has increased significantly over the last decades. Furthermore, modern digital infrastructure (like data banks) collects and provides thousands of protein sequences, while computational and AI‐driven programs help researchers expand the biocatalytic toolbox with respect to environmentally sustainable processes.^[132]^ However, there are still many challenges in modern research concerning enzyme immobilization, developing reaction platforms for flow and multistep biocatalytic reactions, and the use of non‐aqueous media or cofactor recycling systems.[^132^, ^133^]

3D printing offers a solution to these challenges by providing platforms made from low‐residue materials featuring highly specialized geometries and intricate structures that can be readily adjusted to evolving experimental needs.^[134]^ In this chapter, we will explore how 3D printing can elevate biocatalytic research across various domains – including flow chemistry, reaction systems, enzyme immobilization, and the integration of 3D printing of plastics and bioinks (referred to as multi‐material printing).

### 4.1 3D Printing of Reaction Ware

One major advantage of 3D printing for the generation of reaction ware like reactors, mixers, or flow channels lies in the ability to control the contact surface and, therefore, to shape the course of a reaction by producing specialized geometries through CAD design. Surface functionalization techniques can further enhance processes like spatial regulation of mass transport or high‐density immobilization of enzymes.^[135]^ However, to date the adoption of 3D printing within the field of chemistry has been limited, mainly due to a lack of familiarity with CAD design programs. In an attempt to help address this challenge, Hou et al. introduced a tool called “ChemSCAD” (chemical synthesis by computer‐aided design) for developing digital reactors based on chemical operations, physical parameters, and synthetic sequences to produce a given target compound.^[136]^ Built on Python, the software includes pre‐designed modules like tubes, filters, or connectors, requiring much less expertise in CAD design compared to traditional programs like “SolidWorks”.^[136]^

3D printing also has great potential for use in flow biocatalysis. Performing biocatalytic reactions under flow can increase product yield by prolonging the reaction time. Moreover, a series of reactors or parallelization can also be implemented, and reaction conditions such as temperature, pH, pressure, or flow rate can easily be controlled.^[10]^ 3D‐printed microfluidic systems can improve those reactions by allowing high heat transfer, efficient mixing, and enhanced flow capacities.^[137]^

Peris et al. have used 3D printing to develop tunable microbioreactors for transamination reactions using ɷ‐transaminase under continuous flow.^[138]^ The reactor was generated by FDM with nylon as printing material, and the surface was then functionalized (via acidic treatment, followed by glutaraldehyde and PEI) for enzyme immobilization. As a model reaction, the conversion of (R)‐methylbenzylamine into acetophenone under continuous flow was successfully performed.^[138]^

**Figure 3:**
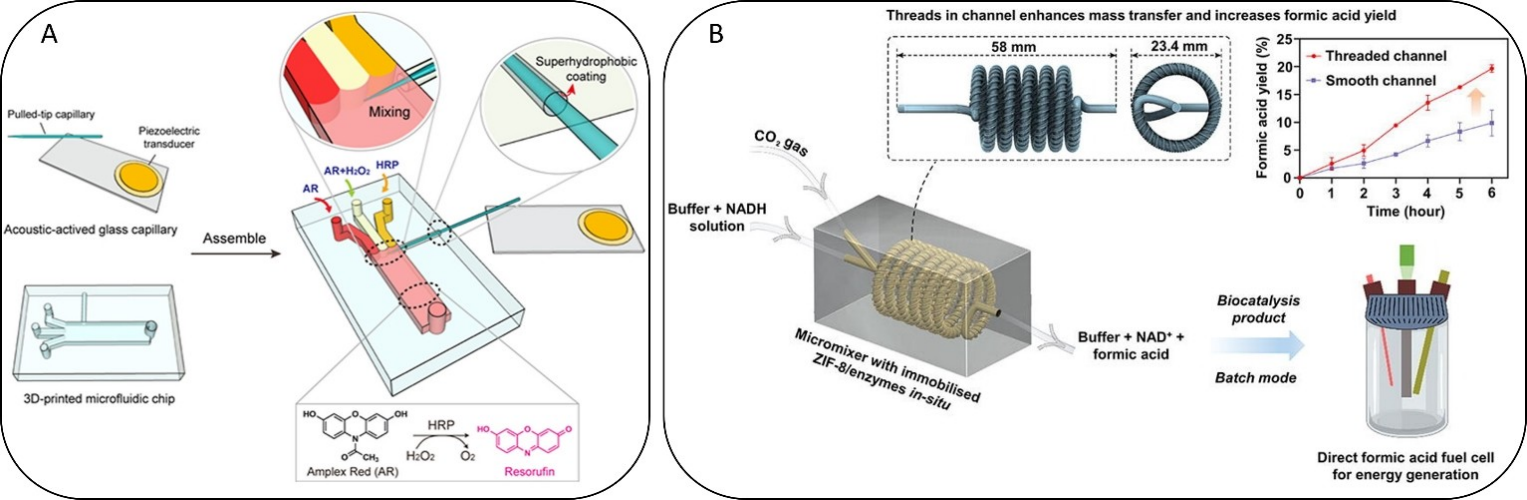
(A) Microfluidic device for enzymatic kinetic measurements. A pulled‐tip glass capillary is inserted into a 3D‐printed microfluidic channel through a side anchoring channel. Vibration is induced on the sharp tip using a piezoelectric transducer. Fluorescence signals resulting from the reactions catalyzed by the HRP enzyme with amplex red and H_2_O_2_ are measured downstream in the channel after complete mixing of all reagents. Adapted with permission from.^[142]^ (B) 3D‐printed biocatalytic helical micromixer biomineralized with an enzyme MOF film including carbonic anhydrase (CA) and formate dehydrogenase (FDH) for the conversion of CO_2_ into formic acid. Through the introduction of threaded channels, an increase in formic acid yield could be achieved, that is later used in a fuel cell for energy generation. Adapted with permission from.^[143]^

Aside from reaction chambers, micromixers play a vital role in microfluidic applications for biocatalysis, because they help to achieve appropriate mixing in an environment that is defined by low Reynold numbers and laminar flow. Micromixers can be described as being either active[^139^, ^140^] (e. g., through acoustic or mechanical mixing) or passive[^49^, ^123^, ^141^] (e. g., without external force for mixing). Li et al. developed a 3D‐printed microfluidic device based on a single vibrating sharp‐tip mixer for one‐step kinetic measurements of horseradish peroxidase (HRP).^[142]^ This device, which was based on polyethylene glycol diacrylate (PEGDA) and 2‐nitrophenyl phenyl sulfide (NPS), enabled the mixing of multiple fluid streams with minimal length (300 μm) and time (3 ms) and a wide range of working flow rates from 1.5 μL min^−1^ to 750 μL min^−1^. Active mixing within the reaction chamber was performed through an external acoustic‐activated glass capillary attached to a piezoelectric transducer (see Figure 3A).^[142]^

Chai et al. went one step further and created a 3D‐printed helical biocatalytic micromixer coated with an enzyme/metal‐organic framework (MOF) thin film (including carbonic anhydrase (CA) and formate dehydrogenase (FDH)) for the conversion of CO_2_ to formic acid.^[143]^ The mixer was printed via SLA, and functionalized with dopamine hydrochloride (PDA) and PEI as a basis for the coating of enzyme‐MOF particles (see Figure 3B). Through the introduction of threaded channels into the micromixer, a 170 % increase in formic acid yield was achieved, demonstrating the importance of mixing for biocatalytic reactions.

3D printing can also be used to produce catalytically active devices, as demonstrated by Kim et al., who developed a density‐adjustable, 3D‐printed, ABS‐based, interfacial device in combination with immobilized CA for the biocatalytic conversion of CO_2_ into bicarbonate (HCO_3_^−^) at gas‐liquid interfaces.^[144]^

Goel et al. used FDM printing of conductive filaments based on carbon black (CB) for the preparation of highly porous multi‐functional electrodes. These electrodes were used for the immobilization of enzymes via EDC‐NHS crosslinking for the generation of a glucose/O_2_ biofuel cell (Laccase for biocathode and GOx for bioanode).^[57]^

Although 3D printing for the generation of reaction ware is still a relatively new application area within the field of biocatalysis, even this handful of recent examples illustrates the great potential of biocatalytic reactions performed through specific systems generated by 3D printing. Due to the development of easy‐to‐handle CAD programs, lower cost of 3D printers, and increase in interdisciplinary research, we fully expect that the advantages offered by 3D printing will be leveraged by researchers to create unique reaction ware for biocatalytic reactions within the next few years.

### 4.2 3D Printing for Enzyme Immobilization

Enzyme immobilization has a rich history in biocatalytic research, primarily as a way of protecting biocatalysts from harsh environmental conditions.^[145]^ Immobilized enzymes exhibit increased resistance to environmental changes and can be much more easily recovered when compared to their free counterparts in solution.^[146]^ Accordingly, immobilization techniques play a vital role in biocatalytic processes. Classic immobilization strategies include techniques such as covalent binding, encapsulation, entrapment, or adsorption.^[146]^

The immobilization of enzymes on 3D‐printed polymers represents a relatively new area of research interest. Specifically, porous networks with high surface‐to‐volume ratios (such as polymers with surface functionalization or bioprinted hydrogels) can be optimized to meet context‐specific needs and thereby offer significant potential for high‐density enzyme immobilization. This capability enables the development of robust, efficient, low‐cost, and long‐term stable processes.^[135]^ Several important parameters must be considered when employing 3D printing for enzyme immobilization, including material selection (particularly for hydrogel composition to withstand reaction conditions and ensure long‐term stability). Additionally, factors like mechanical properties and pore size are crucial to carefully take into account when using porous materials.^[10]^

Various polymers have been investigated as potential platforms for enzyme immobilization in recent years, including commonly used materials like PLA,[^56^, ^147^, ^148^] PET,^[149]^ and Polyamide (PA),[^150^, ^151^] as well as more novel emerging materials such as nanocellulose,[^152^, ^153^] hydrogels,^[154]^ or 3D‐printed ceramics.^[155]^

PLA is one of the most frequently used polymers in 3D printing. Aside from its obvious inherent advantages (biocompatibility and biodegradability), various PLA‐based multi‐component printing materials (including components like conductive polymers^[156]^) are also readily commercially available, making it of particular interest for exploration with respect to enzyme immobilization, especially in the field of electrochemical biosensor fabrication.

Ye et al. investigated the potential of carbon fiber‐reinforced PLA (C‐PLA).^[56]^ Different objects (i. e., cubes, spheres, pyramid shapes, and microfluidic reactors) were designed, printed, and then modified with functional amino groups using wet chemistry techniques. As a proof‐of‐concept study, four enzymes (penicillin G acylase (PGA), protease, glycosidase and lipase) were immobilized using different methods, and their activity and protein recovery were then measured.^[56]^ High reusability and stability for both PGA and glycosidase were observed. Moreover, the modified PLA material also exhibited useful properties like increased roughness and adsorption, high specific surface area, and low cost, giving it tremendous potential as a carrier material.^[56]^

Zhang et al. used PLA‐based 3D‐printed macro scaffolds for the immobilization of lipases for the resolution of racemic 1‐indol via transesterification.^[147]^ The surface was modified in a multi‐step process to introduce phenyl groups (Ph) with different linker lengths as anchor points for enzyme immobilization, leading to increased operational stability while maintaining catalytic activity or stereoselectivity.^[147]^

Aside from Ph‐groups, the use of anchor peptides is another interesting approach to inducing biocatalyst immobilization. When fused to the enzyme sequence, such peptides can function as a “tail” for surface attachment, thus enabling oriented noncovalent immobilization. This technique has been used for several different materials such as PA,^[151]^ PP, carbon, and gold.^[149]^ Büscher et al. used anchor peptides to immobilize phenolic acid decarboxylase (PAD) on a PET surface for the enzymatic decarboxylation of ferulic acid.^[149]^ A major advantage of this approach is the possibility of including spacers between the attaching side of the anchor peptide and the enzyme, thus further enhancing the mobility of the biocatalyst.^[149]^

Beyond the surface modification of plain 3D‐printed polymers, enzyme immobilization can also be performed on 3D‐printed porous scaffolds. In comparison to planar surfaces, porous scaffolds allow for the immobilization of even larger amounts of biocatalysts due to their high surface‐to‐volume ratio.^[157]^

Lackner et al. used nanocellulose‐based 3D‐printed porous scaffolds for the immobilization of glycosyltransferases.^[152]^ Scaffolds were produced by DIW using a three‐component ink based on nano‐fibrillated cellulose (for strength and structural support), carboxymethylcellulose (for interfacial adhesion), and citric acid (for crosslinking). The negative surface charge was used for the co‐immobilization of two different glycosyltransferases harboring a cationic binding molecule via electrostatic attraction. Enzymes were shown to be active in a cascade reaction to achieve the natural C‐glycoside nothofagin from phloretin with 95 % conversion and a maximum reusability of five reaction cycles.^[152]^

Another three‐component bioink – consisting of sodium alginate, polyacrylamide, and hydroxyapatite – was developed by Shen et al. to create a hybrid polymer network hydrogel via 3D bioprinting that was suitable for the *in‐situ* immobilization of GOx and catalase.^[154]^ The inert hydrogel along with its incorporated enzymes demonstrated enhanced catalytic efficiency and operational stability. As a result, a sustained high glucose conversion rate of 97 % was achieved and maintained even after four reaction cycles.^[154]^ Yet another relatively new approach is the use of 3D‐printed ceramics for enzyme immobilization: These inorganic carriers have high chemical, mechanical, and thermal stability, and also boast high surface‐to‐volume ratios which do not pose any risk of unwanted enzyme inactivation.^[158]^

Valotta et al. employed 3D‐printed ceramics as support material for the immobilization of PAD for the decarboxylation of coumaric acid to vinyl phenol (an important active pharmaceutical ingredient (API) precursor) under continuous flow.^[155]^ Various ceramic inserts with different hydraulic diameters and surface‐to‐volume ratios were CAD‐designed, printed via lithography‐based ceramic manufacturing, and then covalently bound with enzymes via NHS‐EDC coupling. A design of experiments (DoE) using different parameters (temperature, flow rate, and dilution ratio) was performed for systematic investigation and process optimization, leading to an 8‐fold increase in product yield compared to previously obtained values for this process.^[155]^ These examples illustrate the wide range of possible applications using 3D printing for enzyme immobilization.

Finally, the combination of different 3D printing materials (e. g., plastics and hydrogels) can potentially be advantageous. For example, a flow reactor can be fabricated via 3D printing of plastics and provide the required stability of the system, while the hydrogel is fabricated inside the reactor and contains the embedded enzyme. Croci et al. developed an agarose‐based hydrogel to entrap enzymes for the reductive amination of benzaldehyde under continuous flow.^[159]^ For the desired reaction, two different enzymes (amine dehydrogenase and FDH) were both co‐entrapped in a hydrogel, which was then integrated into a 3D‐printed, methacrylate‐based flow reactor.^[159]^ This system was used for the reductive amination of benzaldehyde under continuous flow over 120 h, leading to an analytical yield of 47 %.

Schmieg et al.^[160]^ entrapped three different enzymes (ADH, benzoylformate decarboxylase and ß‐galactosidase) in a PEGDA‐based hydrogel which was embedded into an Inkjet‐printed reactor. Enzymatic activity was measured and compared to that of the free enzyme – but in this case, only effectiveness factors between 6 %–14 % could be attained.^[160]^ These findings underscore the significant challenges still posed by mass transfer limitations within hydrogel‐based systems, where the performance of the entrapped enzymes depends not only on the individual activity loss due to immobilization but also on the equilibrium achieved between reaction rate and mass transfer within the bioprinted hydrogels.^[161]^ To create highly catalytically active systems, optimization of the pore size of the utilized hydrogel and printing parameters such as strand distance is essential. The integration of hydrogels into 3D‐printed polymer‐based reactors is a relatively easy‐to‐use and straightforward process which can be used to eliminate (often toxic) multi‐step and wet‐chemistry‐based surface functionalization techniques.

Besides the use of 3D‐printed hydrogels for enzyme incorporation – even catalytically active living materials such as bacteria[^162^, ^163^, ^164^] or yeast[^165^, ^166^, ^167^] – have been embedded into hydrogels. Also, the co‐cultivation of different microorganisms in a hydrogel system has been demonstrated, providing the benefit that these organisms can work synergistically together to produce various components.^[168]^ Extensive publications on this topic have been published in recent years,[^169^, ^170^, ^171^, ^172^, ^173^] discussing different 3D printing techniques for cell‐embedding into hydrogels as well as the advantages and disadvantages of various bioinks for microbial and cellular biocatalytic applications. In general, hydrogels can provide an ideal environment for living cells due to their biocompatibility, tunable properties, and high water content, allowing the influx of metabolites and the efflux of waste.^[169]^ By using of modern 3D printers, precise geometries can be achieved to optimize surface area, mass transfer, and overall reaction efficiency. Yet, it should be noted that the challenge remains to cause only minimal damage to living organisms during the bioprinting process.^[169]^

By combining multiple materials with genetically engineered microorganisms, a broad range of biocatalytic reactions is feasible.^[169]^ Examples of catalytically active living materials include the work of Saha et al., who employed DIW to produce a dimethylacrylate‐based hydrogel to incorporate yeast cells for the fermentation of glucose to ethanol^[165]^ and Cui et al., who incorporated *Streptococcus zooepidemicus* in a 3D‐printed hydrogel for the production of hyaluronic acid (HA).^[164]^

## 5 3D Printing for Biosensing Applications

3D printing for the development of biosensors is a dynamically evolving field in life sciences and biochemistry. It can either be used for device fabrication (e. g., for microfluidic flow cells) to integrate commercially available sensors, or for the intrinsic printing of an entire device. It thus provides an attractive alternative method for fabricating transducers, aiming to design smaller, faster, and more efficient devices that are easily accessible with low sample consumption and high‐cost effectiveness.[^7^, ^52^] Through the integration of multiple components (with tailored geometry and shape) in a single device, customized sensor arrays capable of simultaneously detecting multiple analytes can be produced.^[174]^ Various articles have been published in recent years focusing on electrochemical sensors and the combination of electric conductive materials with 3D‐printed polymers.[^175^, ^176^, ^177^] These studies mainly deal with sensors that do not rely on specific capture elements like antibodies or enzymes for biological analyte detection. Accordingly, here we provide an overview of recent research (mainly published within the last four years) focusing on capture element‐based biosensors in combination with 3D printing.

### 5.1 Aptamer‐Based Biosensors

Aptamers – often referred to as chemical antibodies – are single‐stranded DNA molecules (ssDNA) that can bind various targets with high specificity. Their distinctive advantages (including small size, low cost, uniform synthesis, and customized modifications) make them an ideal capture probe for deployment within biosensing applications.^[178]^ Various aptasensors for malaria,^[26]^ cancer,[^179^, ^180^] protein,[^181^, ^182^] bacteria,^[48]^ and virus^[183]^ detection have already been integrated into 3D‐printed devices, and will be discussed in the following section. Malaria remains a tremendous global burden in the field of health care, with an estimated caseload of ~250 million in 2022 alone.^[184]^ Within the field of malaria diagnosis, Fraser et al. developed a portable microfluidic aptamer‐tethered enzyme capture (APTEC) biosensor.^[26]^

**Figure 4:**
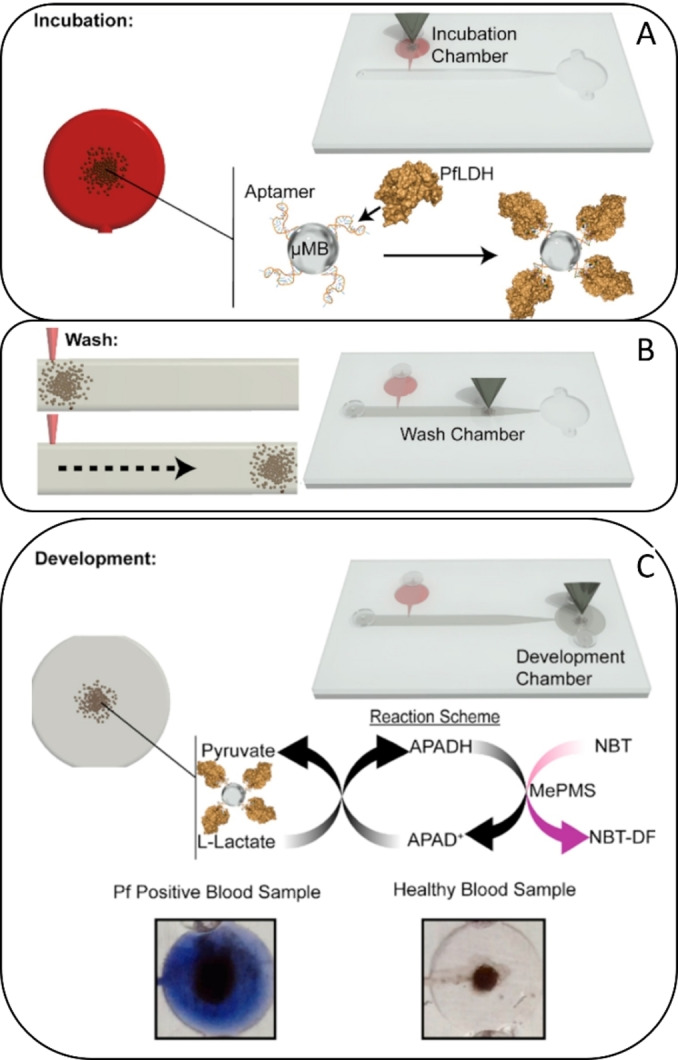
(A) 3D‐printed microfluidic device for the detection of malaria biomarker PfLDH. Aptamer coated micro‐magnetic beads (μMBs) are incubated with human blood samples inside an incubation chamber, followed by magnetically moving to the washing chamber (B) to separate them from nonspecific blood components. (C) The signal is generated in a development chamber, where positive samples turn purple by the formation of an insoluble formazan dye (NTB‐DF). Adapted with permission from.^[26]^

Aptamer‐coated magnetic microbeads (μMBs) were used to capture the malaria biomarker plasmodium falciparum lactate dehydrogenase (PfLDH). The SLA‐printed microfluidic device consisted of three separate chambers (incubation, washing and development), and it only required a few microliters of sample and reagents. It was used for the colorimetric detection of PfLDH from *in vitro* cultured malaria samples, as well as clinical samples obtained from malaria patients (see Figure 4).^[26]^

In the field of bacteria detection, Siller et al. developed a polyacrylate‐based 3D‐printed microfluidic flow cell combined with aptamer‐modified screen‐printed electrodes (SPEs) for the detection of *E. coli* via electrochemical impedance spectroscopy (EIS).^[48]^ A micromixer^[49]^ for enhanced sample homogenization was integrated, and the system allowed for label‐free detection of pathogenic *E. coli* under dynamic flow with high sensitivity and specificity. The sensor was also reusable; it could be flushed out with boiling water to remove *E. coli*, followed by a refolding of the aptamer in a specific buffer.^[48]^

Due to the global COVID‐19 pandemic, research concerning biosensors suitable for virus detection increased dramatically. For example, an electrochemical membrane‐based aptasensor in combination with a 3D‐printed flow cell for the detection of the SARS‐CoV‐2 receptor binding domain (RBD) was developed by Tabrizi et al.^[183]^ This sensor relied on a functionalized nanoporous anodic aluminum oxide membrane (NPAOM), decorated with AuNPs as anchor points for aptamer immobilization. The binding of thiolated aptamers was performed inside the microfluidic sensor chamber under flow. The system demonstrated a low limit of detection (LOD) (ng mL^−1^ range) and high selectivity even in the presence of interfering agents like hemagglutinin and neuraminidase from the influenza A virus.

Furthermore, no change in biosensor performance was observed after 21 days, indicating that the aptasensor was also highly stable.^[183]^

The detection of protein markers can be used to reveal neurodegenerative diseases such as Alzheimer's – a disease which is estimated to triple in prevalence by 2050.^[185]^ Not surprisingly, research is now increasingly being directed towards developing biosensors suitable for Alzheimer's biomarker detection (e. g., beta amyloids), which can potentially provide early treatment and enable physicians to slow the progression of the disease. One electrochemical aptasensor for the detection of beta‐amyloids based on a 3D‐printed platform integrated with leaf‐shaped gold nano dendrites was developed by Negahdary et al.^[186]^ This publication stands out due to its surface treatment of the 3D‐printed CB/PLA electrode. CO_2_ laser ablation was used to increase the electric conductivity (by removing polymeric material from the electrode surface), followed by the electrodeposition of gold nano dendrites. Thiolated aptamers were immobilized via thiol gold bonding. This biosensor could also be reused six times with a low LOD in the fg mL^−1^ range for beta‐amyloids.^[186]^

### 5.2 Antibody‐Based Biosensors

In addition to aptamers (which are still a relatively new form of biological capture element), monoclonal antibodies (mAbs) are also widely used in point‐of‐care diagnostics (e. g., for ELISAs or paper‐based rapid SARS‐CoV‐2 testing) due to their target specific binding in combination with well‐established industrial production processes.^[187]^ Their use in combination with 3D‐printed reaction ware is a similarly rapidly growing area of interest, with numerous systems published in recent years aimed at facilitating the analysis of different targets with a focus on cancer biomarkers^[188]^ and viruses.[^189^, ^190^]

**Figure 5:**
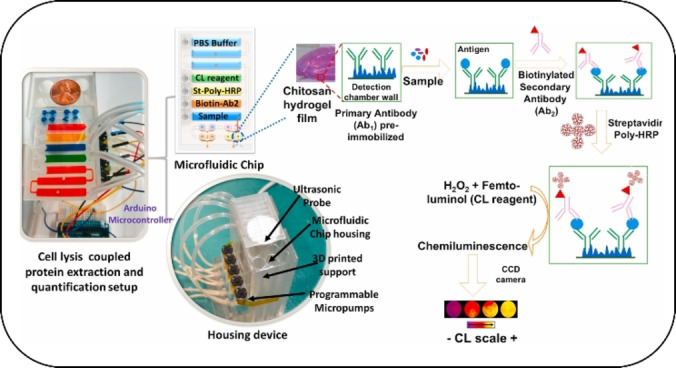
Microfluidic device for cancer detection. The design consists of a microfluidic chip with five inlets connected to peristaltic micropumps, sample and rectangular prism reagent chambers for delivery of sample and reagents to the detection compartment. The assay protocol utilizes poly‐HRP and femto‐luminol to produce chemiluminescence that is captured via a CCD camera. The chip is mounted on the housing device support equipped with sonic lysis probe and micropumps that are controlled by an Arduino microcontroller. Adapted with permission from.^[191]^

For instance, a microfluidic sandwich ELISA for the chemiluminescence‐based detection of neck squamous cell carcinoma (HNSCC) metastasis, accompanying protein biomarkers (desmoglein 3 (DSG3), and vascular endothelial growth factors‐A/C (VEGF−A/C), was developed by Sharafeldin et al.^[191]^ This PA‐based, SLA‐printed microfluidic device was capable of lysing cells through a 2 s ultrasonic pulse and quantifying released proteins in a reaction chamber with a LOD in the fg mL^−1^ range. Capture antibodies immobilized onto inner walls coated with a highly swollen 3D chitosan hydrogel film were used for the sensing system (see Figure 5). This system illustrates the great potential of 3D printing for device fabrication, since different printing materials (hydrogels and polymers) as well as reaction ware (cell disruptor) were all successfully integrated into one device, thus combining several different steps that would have had to be performed separately in a regular analysis.

**Figure 6:**
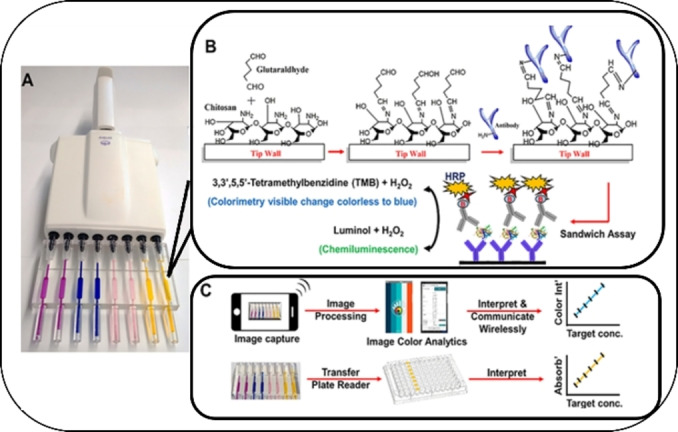
(A) 3D‐printed sandwich immunoassay ELISA tips loaded with food dyes. (B) Inner walls of the transparent material, functionalized with chitosan and glutaraldehyde for the covalent immobilization of capture antibodies followed by sandwichimmunoassay and signal measurement for colorimetry and CL. (C) Signal capture and processing flow using smartphone or microplate reader. Adapted with permission from.^[188]^

Also, antibody‐functionalized pipette tips were fabricated for the ELISA‐based detection of cancer biomarkers.^[188]^ The inner walls of the SLA‐printed pipette tips were modified with chitosan hydrogels and glutaraldehyde to affect covalent immobilization of capture antibodies (see Figure 6B); the reagents were then moved in and out by pipetting (see Figure 6A). Signals were generated using colorimetric or chemiluminescent reagents, and could be quantified by a cell phone, CCD camera, or plate reader (see Figure 6C). The system was put to the test with the simultaneous detection of four different cancer biomarker proteins in human spiked serum. LODs in the pg mL^−1^ range – a range similar or lower compared to that achieved using commercial microplate ELISAs – were reported.^[188]^

Once again, due to the COVID‐19 pandemic, most 3D‐printed antibody biosensors that have been published over the last four years have arisen in the subfield of virus diagnostics,[^192^, ^193^] in combination with new printing techniques such as Aerosol Jet Printing (AJP),[^189^, ^194^] and/or conductive materials like graphene/PLA.[^190^, ^195^]

Ali et al. utilized AJP in conjunction with reduced graphene oxide (rGO) nanoflakes to create a platform for immobilizing specific viral antigens and thereby detecting COVID‐19‐induced antibodies.^[189]^ The three‐dimensional electrodes that were generated were incorporated into a microfluidic device, enabling the detection of antibodies to the SARS‐CoV‐2 spike S1 protein through a smartphone‐based user interface. In addition to specific detection, the sensor could also be regenerated within a minute using a low‐pH chemistry to elute the antibodies from the antigens, allowing the same sensor to be reused to test multiple samples. Another SARS‐CoV‐2 biosensor that incorporates graphite within a polymer matrix of PLA was developed by Stefano et al.^[190]^ The four‐step procedure involves mixing graphite and PLA in a heated reflux system, followed by recrystallization, drying, and extrusion. With this technique, an active biosensing material without any further need for surface activation was developed and modified with mAbs against SARS‐CoV‐2 spike protein via EDC/NHS coupling. This innovative sensor design achieved a LOD in the nmol mL^−1^ range.

### 5.3 Enzyme‐Based Biosensors

Enzymes have a long history of being used as recognition elements for biosensors. Indeed, the first reported system for the detection and quantification of glucose using GOx was published by Clark and Lyons in 1962.^[196]^ Since then, many improvements have obviously been made to increase both the stability and the sensitivity of enzyme biosensors. In recent years, the use of 3D printing in combination with enzymes for biocatalytic applications has become an increasingly popular approach (e. g., to produce enzymatic fuel cells or to provide specific surfaces for enzyme immobilization). Regarding the earlier‐mentioned biocatalytic applications, enzyme‐based biosensors are often generated in combination with 3D printing rather than being implemented into 3D‐printed reaction ware. Most of the published systems generated with this approach are in the field of glucose biosensing, since this is the most established application in the field of enzymatic biosensing. A number of intriguing systems suitable for glucose detection have recently been reported,[^35^, ^197^, ^198^, ^199^] that mostly rely on FDM printing and conductive carbon or modified graphene/PLA (G‐PLA) inks.

For example, Cardoso et al. developed a biosensor capable of the simultaneous detection of glucose, uric acid, and nitrite in blood plasma.^[35]^ They used the oxygenated groups on G‐PLA for GOx immobilization by crosslinking with glutaraldehyde, achieving LODs in the μmol L^−1^ range.

A similar approach was developed by Wang et al.^[197]^ Their FDM‐printed nanocarbon electrode was electrochemically activated by incubation with dimethylformamide (DMF), followed by washing with acetone/ethanol and drying at 50 °C. GOx was then covalently bound through EDC/NHS coupling, for the detection of H_2_O_2_ and glucose by chronoamperometry with LODs in the μM range.

An interesting example of a smartphone‐operated 3D‐printed electrochemical luminescence (ECL) biosensor was reported by Bhaiyya et al.^[198]^ They used luminol/H_2_O_2_‐based enzymatic reactions for the selective sensing of glucose and choline in real blood serum. The device was FDM‐printed using graphene/PLA, and then activated by DMF treatment.

Besides the detection of glucose, which is one of the best‐established approaches within enzymatic biosensing, several systems based on enzyme‐modified, FDM‐ or SLA‐printed, electrochemically activated graphene/PLA electrodes have already been published for the analysis of various molecules, such as serotonin,^[36]^ hydrogen peroxide,^[200]^ cholesterol,^[201]^ and chiral amino acids.^[202]^

Activation of the mentioned biosensing electrodes often relies on the use of DMF or mechanical scrubbing in combination with washing steps (using organic solvents like ethanol, acetone, or Isopropanol). Electrochemical activation can also be performed via enzymatic digestion of the respective electrode, rather than by using chemical treatment. Manzanares‐Palenzuela et al. used proteinase‐K for surface activation of 3D‐printed graphene/PLA electrodes.^[203]^ The enzymatic digestion resulted in exposed graphene sheets within the PLA structure that led to tailorable electrode performance. As a proof‐of‐concept, they immobilized the enzyme alkaline phosphatase via physical adsorption onto the activated surface for the electrochemical detection of 1‐naphthol.

**Figure 7:**
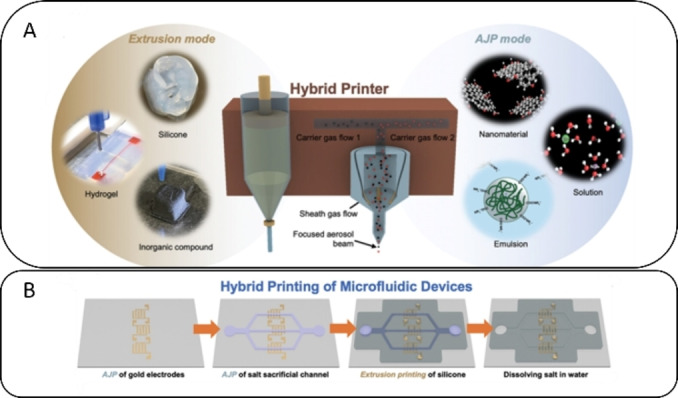
Hybrid printing of microfluidic devices. (A) Schematic illustration of the multi‐material, multi‐scale hybrid printing method integrating extrusion printing and AJP. (B) Printing process for the fabrication of complete microfluidic devices. After AJP of the respective gold electrodes and salt channels, extrusion mode is used for the printing of silicone. Adapted with permission from.^[38]^

An ideal biosensor fabrication would consist of a fully printed device that combines different printing techniques (hybrid printing), thus requiring no additional functionalization or preparation steps. A recently interesting approach using hybrid printing for biosensor fabrication was published by Du et al.^[38]^ They combined a multi‐material AJP head featuring dual ink atomizers with an EP‐based DIW head to create a microfluidic biosensor that was fully printed and required no additional assembly. This biosensor included embedded fluidic channels and functionalized electrodes, achieving sub‐100 μm spatial resolution for amperometric sensing of lactate in sweat. The DIW head is capable of printing viscous polymer inks to produce complex 3D structures, whereas the 3D conformal AJP head can print water‐soluble sacrificial inks to form microfluidic channels and functionalized microelectrode arrays for biosensing (see Figure 7). Lactate was detected in sweat by EDC/NHS immobilized lactate oxidase (LOx) onto AuNPs, with a LOD in the nM range. The authors also developed customized printing software to modulate the printing parameters such as sheet gas flow rate (SGFR) and carrier gas flow rate (CGFR) as well as printing speed, nozzle size and number of layers during the printing process.

## 6 Summary and Outlook

In this review, we have set out to offer some insights into promising recent developments in 3D printing for applications in the fields of biocatalysis and biosensing. Modern 3D printing is unquestionably a versatile technology, and as such, many different methods and materials (with various advantageous and/or potentially problematic properties) come into play. Due to its straightforward use, its relatively easy implementation, and the feasibility of leveraging it to achieve rapid prototyping simply based on modifying CAD designs, 3D printing is an increasingly attractive technique that is being used in ever more applications, such as the integration of biosensors into customized flow cells or the production of hydrogels and porous scaffolds for biocatalysis. Yet, these new applications also bring along new challenges: The reproducible and leakage‐free integration of biosensors into 3D‐printed microfluidic systems is challenging and different approaches exist (e. g., magnets,^[48]^ gluing,^[181]^ adhesive tapes^[182]^) as for each 3D printing material and biosensor, the most suitable integration method needs to be found. However, in terms of optical sensor integration, 3D printing might partially present the solution already. For instance, two‐photon polymerization allows the fabrication of optical microstructures which could be directly printed inside microfluidic systems – providing a new approach to solving leakage issues and increasing the reproducibility of optical sensor integration.

Moreover, in the future, it will be crucial to translate new biosensor concepts from the laboratory to “real‐world” applications and wearable biosensors are broadly acknowledged to fill this gap by providing non‐invasive and convenient healthcare monitoring^[204]^ and point‐of‐care^[205]^ testing solutions. Also, in this emerging biosensor field the contribution of 3D printing in current and future research is unquestionable as emphasized by Parupelli et al. in their review article.^[206]^

In biocatalysis, the entrapment or immobilization of enzymes goes along with the challenge of diffusion limitation^[207]^ which often slows enzyme kinetics and impairs conversion rates. This is not only true for 3D‐printed materials, but also for traditional enzyme entrapment methods.^[208]^ Yet, 3D printing allows to design and print customized carrier matrices where different geometries can be created and adapted to the individual kinetics of a respective enzymatic reaction. Thus, 3D printing can indeed be the future technique to improve the efficiency of entrapped or immobilized enzymes.^[207]^

## Conflict of Interests

The authors declare no conflict of interest.

7

## Biographical Information

*Jonathan Nyenhuis is a PhD student at the Chair of Technical Biology, Institute of Physics, University of Augsburg. He obtained his Master of Science in Life Science at the Leibniz University Hannover at the Institute of Technical Chemistry. His current research focuses on the development of optical biosensors in combination with 3D printing for applications in bioprocess monitoring*.

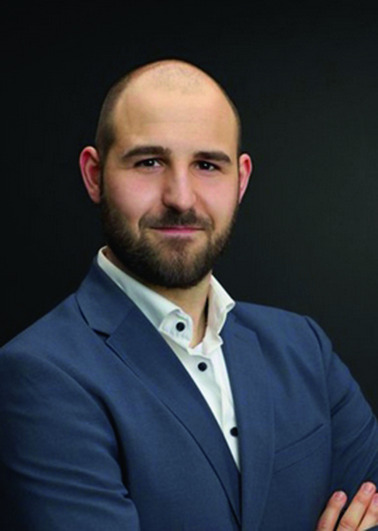

## Biographical Information

*Dr. Christopher Heuer is a Postdoctoral Researcher at the Chair of Technical Biology, Institute of Physics, University of Augsburg. He studied Life Science at Leibniz University Hannover (LUH) and obtained his PhD in Biotechnology within a dual doctorate program at LUH and the Technion – Israel Institute of Technology. His current research focuses on the application of miniaturized and microfluidic 3D‐printed systems for a wide variety of applications ranging from bioanalytics to biosensors for bioprocess monitoring and point‐of‐care diagnostics*.

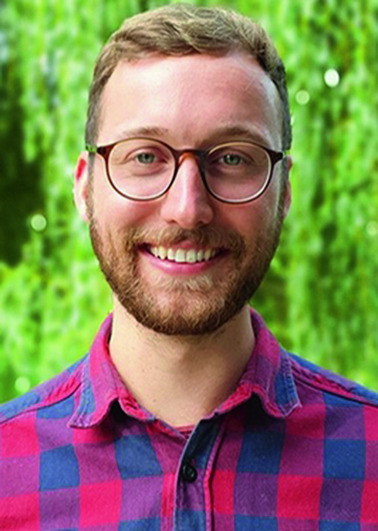

## Biographical Information

*Prof. Dr. Janina Bahnemann is a Full Professor and Head of the Department “Technical Biology” at the University of Augsburg since 2022. She studied Life Science at Leibniz University Hannover and obtained her PhD from Hamburg University of Technology. In 2015 she joined the California Institute of Technology (USA) as a postdoc. Afterwards, she led an independent Emmy Noether (DFG) research group at Leibniz University Hannover (2017–2022) and held a substitute professorship at Bielefeld University (2021–2022). Her research focuses on cell culture and microsystems technology and the development of biosensors for biomedical and biotechnological applications*.

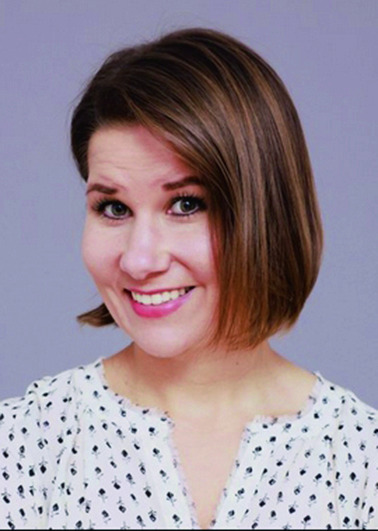

## Data Availability

Data sharing is not applicable to this article as no new data were created or analyzed in this study.
